# A conserved bacterial signature characterizes plant microbiome responses to drought

**DOI:** 10.3389/fmicb.2026.1768028

**Published:** 2026-03-27

**Authors:** Bianca-Maria Cosma, Thomas Abeel

**Affiliations:** 1 Delft Bioinformatics Lab, Delft University of Technology, Delft, Netherlands; 2 Infectious Disease and Microbiome Program, Broad Institute of MIT and Harvard, Cambridge, MA, United States

**Keywords:** 16S, drought, microbiome, plant resilience, soil

## Abstract

**Introduction:**

Plant-associated microbes contribute to host resilience under stress, yet the extent to which microbial responses to drought generalize across hosts and environments remains unclear.

**Methods:**

Here, we performed a meta-analysis of 13 studies including more than 3,000 root and bulk soil samples from 52 plant hosts to identify bacterial taxa consistently affected by drought and link them to inoculation outcomes. Using a standardized processing workflow and differential abundance analysis, we derived a “drought signature” of taxa differentially abundant under water limitation across the endosphere, rhizosphere, and bulk soil.

**Results:**

The signature is dominated by Gram-positive Actinobacteria enriched under drought, including *Kribella*, and by Gram-negative taxa depleted under drought, such as *Ramilbacter*. Comparison with four independent inoculation experiments revealed limited overlap between drought and inoculation responses, with only *Nitrospira* depleted in both contexts.

**Discussion:**

Studies reporting improved plant performance under inoculation also exhibited stronger and more extensive microbial shifts, suggesting that the magnitude of community restructuring may be characteristic of successful inoculation outcomes. This work provides the fi rst genus-level meta-analysis of microbial responses under drought and inoculation, across hosts and experimental conditions, and delivers a unifi ed root and soil microbiome dataset.

## 1 Introduction

Different terms may be used to describe plants and their associated microbes, reflecting trends in human health research (Grice and Segre, 2012; Postler and Ghosh, 2017; Van De Guchte et al., 2018). Microbes may be called a plant's “second genome” (Berendsen et al., 2012), and the plant–microbe symbiotic unit is sometimes referred to as a “holobiont” (Margulis, 1991; Rosenberg et al., 2009; Vandenkoornhuyse et al., 2015). Soil and root microbes exemplify these concepts, as they help plants fight disease and pests (Pieterse et al., 2014; Collinge et al., 2022), acquire nutrients such as nitrogen (Trivedi et al., 2020) and phosphorus (Rodríguez and Fraga, 1999), and tolerate abiotic stressors like drought (Aslam et al., 2022; Igiehon and Babalola, 2021). Trivedi et al. (2022) even predicted that short-term plant responses to climate change will stem mainly from shifts in the plant microbiome, owing to microbes' rapid evolutionary dynamics.

Unsurprisingly, the plant microbiome as a sustainable approach in agriculture is recently gaining traction (Compant et al., 2025). Climate change continues to affect populations across the globe, with the most vulnerable carrying the heaviest burden (IPCC, 2023; Erian et al., 2021). Across 18 countries, over 90 million people are currently facing what the Food Security Information Network (FSIN) defines as “acute food insecurity”: a condition in which access to food is disrupted, threatening the lives or livelihoods of those affected (FSIN and GNAFC, 2025). So, even though farming already contributes to sizable freshwater withdrawals, soil health degradation (FAO, 2021), and greenhouse gas emissions (IPCC, 2023), agricultural production must still expand to meet rising global food demands.

So far, most successful microbial products in agriculture function as nitrogen-fixing bio-fertilizers (O'Callaghan et al., 2022; Compant et al., 2025), with success stories including several *Azospirillum* and *Rhizobium* strains (Chen et al., 2021). Bio-stimulants, another class of microbial products used in agriculture, do not directly supply nutrients but instead enhance plant growth and tolerance against abiotic stressors such as drought. In a meta-analysis, including mostly greenhouse studies, Rubin et al. (2017) reported that inoculated plants had 40% higher yields under drought. But greenhouse and lab conditions often fail to translate to the field, and the sector lacks large-scale microbial solutions against drought (O'Callaghan et al., 2022; Compant et al., 2025). Multiple factors constrain field performance; for example, native microbes frequently out-compete introduced strains (Liu et al., 2023, 2022), and studies still fail to capture generalizable microbial responses to drought across experimental conditions and host species. While some work identifies core drought microbes across hosts (Naylor et al., 2017; Fitzpatrick et al., 2018), others argue that a “one-size-fits-all” approach to microbiome engineering for drought tolerance will not work, because microbial communities differ between plant species and taxa are not consistently selected across hosts exposed to drought stress (Bandopadhyay et al., 2024).

Recent strategies—transplants of soils, whole microbial communities, or sub-communities—seek to “borrow” resilience from drought-tolerant plants and their associated dry soils (Chen et al., 2021; Compant et al., 2025). This falls in line with recent successes in inoculation with synthetic communities (SynComs) to boost drought tolerance (Armanhi et al., 2021; Hu et al., 2021; Francioli et al., 2025), and the belief that more complex microbial communities may have greater chances of success in live soil compared to single strains (Naylor and Coleman-Derr, 2018; Liu et al., 2023). But the performance of such new methods remains unconvincing. Some inoculant communities improve crop performance under drought (Moore et al., 2023; Zhang et al., 2022), while others show no clear benefit (Munoz-Ucros et al., 2022; Swift et al., 2025); the reasons for this variability remain unclear, as does the link between inoculants and microbial drought responses.

Therefore, the aim of this study is to better characterize the drought microbiome, and determine whether microbial taxa affected by drought also play a role in inoculation success for drought-stressed plants. We followed a two-step approach. First, we performed a large-scale analysis of the drought microbiome (including nine studies: Bandopadhyay et al., 2024; Santos-Medelĺın et al., 2017, 2021; Azarbad et al., 2020, 2022; Simmons et al., 2020; Xu et al., 2018; Naylor et al., 2017; Fitzpatrick et al., 2018) and determined a set of representative microbes that are differentially abundant under drought, across studies and plant hosts. The concept of such a “drought signature” was inspired by “disease signatures” in recent studies on the human microbiome (Sun et al., 2024; Li and O'Toole, 2024). In the second step of our approach, we characterized bacterial communities in inoculation experiments with different outcomes, using data from four studies (Moore et al., 2023; Zhang et al., 2022; Swift et al., 2025; Munoz-Ucros et al., 2022). We contextualized these results by linking back to our drought signature. This work is novel in three main aspects: (1) the first large-scale analysis of the root and soil drought microbiome across multiple studies; (2) a processed root and soil microbiome dataset including count tables for 3,282 samples, 13 studies (Bandopadhyay et al., 2024; Santos-Medelĺın et al., 2017, 2021; Azarbad et al., 2020, 2022; Simmons et al., 2020; Xu et al., 2018; Naylor et al., 2017; Fitzpatrick et al., 2018; Moore et al., 2023; Zhang et al., 2022; Swift et al., 2025; Munoz-Ucros et al., 2022), and 52 plant host genotypes; and (3) a comparison of inoculation studies (Moore et al., 2023; Zhang et al., 2022; Swift et al., 2025; Munoz-Ucros et al., 2022) with different outcomes, based on processed data from bulk soil, rhizosphere, and endosphere samples.

## 2 Materials and methods

**Figure 1:**
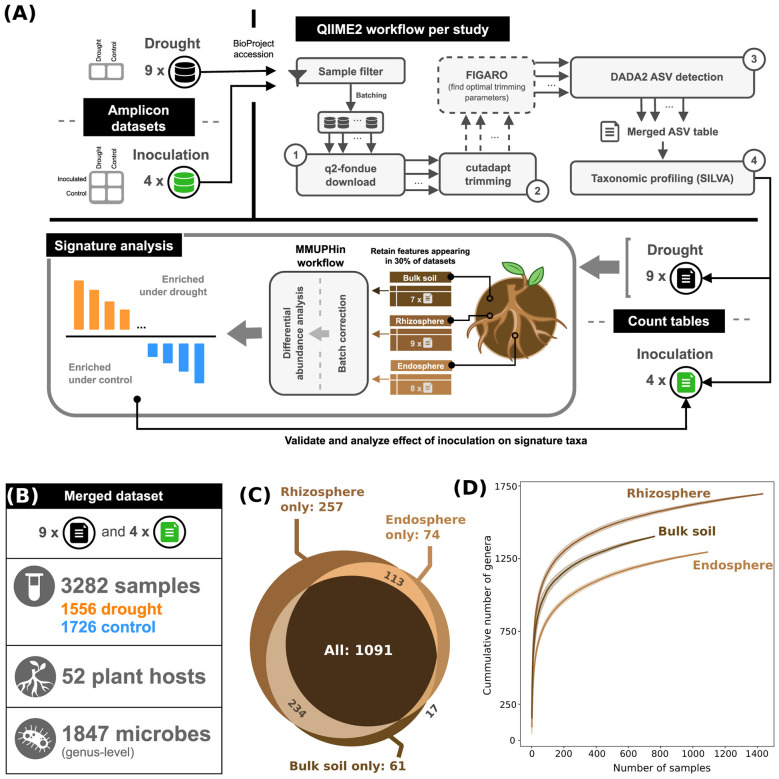
Meta-analysis overview. **(A)** Samples were collected from 13 datasets, with two factorial designs. Amplicon reads in each study were filtered, batched and then processed using QIIME2 (Bolyen et al., 2019). Count tables for the nine drought studies were separated based on soil/root compartment, and differential abundance analysis was performed on a batch-corrected dataset. Drought signature taxa were then analyzed separately in the inoculation experiments. **(B)** Merged dataset statistics. Merging was performed using a union of features (taxa) across studies. **(C)** Venn diagram of microbial genera shared between compartments. **(D)** Rarefaction curves for the cumulative number of genera in three compartments, as a function of the number of samples.

Figure 1A illustrates a high-level overview of our pipeline. We started with 13 amplicon sequencing datasets including root and soil samples (Section 2.1), and processed each study individually using QIIME2, to produce genus-level taxonomic profiles (Section 2.2). We then input these profiles to a downstream analysis step, in which we extracted enrichment and depletion patterns of microbial behavior under drought, and linked them to inoculation responses (Section 2.3).

### 2.1 Amplicon data

#### 2.1.1 Data for drought signature extraction

To determine which bacteria are differentially abundant under drought, we identified nine studies (Bandopadhyay et al., 2024; Santos-Medelĺın et al., 2017, 2021; Azarbad et al., 2020, 2022; Simmons et al., 2020; Xu et al., 2018; Naylor et al., 2017; Fitzpatrick et al., 2018) in which microbial communities were extracted from plants under drought, as well as a control group consisting of plants under normal watering conditions. For consistency, we limited our search to experiments that used Illumina 16S paired-end amplicon sequencing to extract microbial DNA. In addition to sequencing platform and marker gene, we required that studies provide sufficient sample-level metadata to enable stratification by treatment (drought vs. control) and by compartment (rhizosphere, endosphere, or bulk soil). We further restricted inclusion to datasets comprising more than 100 samples. Finally, we only kept studies for which our processing pipeline produced high-quality taxonomic profiles at the genus-level; datasets with high proportions of unassigned taxa (>50% within a sample) were excluded.

#### 2.1.2 Data for inoculation experiments

To analyze how root microbial communities are affected by inoculation, we selected four studies (Moore et al., 2023; Swift et al., 2025; Zhang et al., 2022; Munoz-Ucros et al., 2022) that implemented such an experimental approach. The selected studies also profiled root bacterial communities using DNA amplicon sequencing (16S rRNA). We considered study inclusion based on two criteria. The first was that the study should include at least a 2 × 2 factorial experiment, in which one factor was inoculation (at least two different inocula apart from the sterilized control soil) and the other was water treatment (drought and normal watering conditions). The second criterion was that the study assessed plant performance under all experimental conditions. Metrics to assess plant performance differed per study, and not all studies published their complete metadata, making a direct comparison infeasible. Therefore, based on what the authors reported in the original manuscripts, we identified the experiments by Moore et al. (2023) and Zhang et al. (2022) as successful inoculation experiments, and those performed by Swift et al. (2025) and Munoz-Ucros et al. (2022) as non-successful experiments. Experiments were reported as successful by the original authors when inoculated plants performed better under drought. To simplify analysis, we defined “dry” and “reference” inocula per study, as outlined in Supplementary Table S1. We note, however, that studies tested different inocula, and in some cases they did not perfectly align with a “dry” and “reference” split. For instance, in the study of Moore et al. (2023) both inocula come from stressed environments; in this case, we chose the inoculum that lead to increased plant performance as the “dry” inoculum.

Supplementary Table S1 lists all processed studies and their accessions. Table 1 provides a summary of sample sizes for each study, with a more detailed breakdown available in Supplementary Table S2. Filtering rules were configured per study and are available in our GitHub repository.

**Table 1:** Summary of sample sizes for the studies used in our meta-analysis.

Study	Initial	After filtering	Drought	Control
Drought studies				
Xu et al. (2018)	941	652	194	458
Simmons et al. (2020)	366	161	58	103
Fitzpatrick et al. (2018)	595	302	163	139
Azarbad et al. (2020)	950	292	218	74
Santos-Medelĺın et al. (2021)	612	487	280	207
Bandopadhyay et al. (2024)	306	199	99	100
Naylor et al. (2017)	880	434	193	241
Santos-Medelĺın et al. (2017)	432	197	92	105
Azarbad et al. (2022)	1,331	367	173	194
Inoculation studies				
Moore et al. (2023)	53	39	17	22
Swift et al. (2025)	535	60	25	35
Zhang et al. (2022)	98	40	20	20
Munoz-Ucros et al. (2022)	102	52	24	28

For a more detailed overview, see Supplementary Table S2.

### 2.2 QIIME2 workflow per study

We ran a processing pipeline based on QIIME2 (Bolyen et al., 2019) for amplicon data, v2024.10. This consisted of four steps: (1) NCBI download with q2-fondue (v2022.11.0) (Ziemski et al., 2022); (2) primer removal with Cutadapt (Martin, 2011); (3) trimming and detection of amplicon sequence variants (ASVs) with DADA2 (Callahan et al., 2016); and (4) taxonomic profiling of ASVs with QIIME2's Naive Bayes classifier, pre-trained on SILVA 138, with 99% OTU similarity and full-length sequences.^1^ All steps were performed on batches of 50 samples (batches with fewer than 15 samples were merged with the preceding batch).

We used default parameters for all QIIME plugins, unless explained otherwise.

Between steps 2 and 3, we used FIGARO v1.1.2 to determine truncation and trimming parameters for DADA2 (Sasada et al., 2020) per study; calculated as the median values across batches. For studies where FIGARO failed, we truncated all reads by removing 10% of the amplicon length reported in the studies. When that approach also failed, we used the parameters specified by the authors of the study. For a full list of parameters per study, see Supplementary Table S3.

In step 4, we filtered out amplicon sequence variants (ASVs) and samples as follows. We kept ASVs assigned at least at phylum level and we excluded taxon names that contained the following keywords: Chloroplast, Mitochondria, Eukaryota, Unassigned, Unclassified, Archaea. We also removed samples with a total ASV count less than 10,000.

### 2.3 Downstream analysis

#### 2.3.1 Dataset merging

Separately per compartment (endosphere, rhizosphere, bulk soil), as well as across all compartments, we merged studies by taking the union of taxonomic features across studies. Only for the signature analysis and batch correction, we merged just the nine drought datasets, per compartment, at genus-level, using a stricter integration of the feature space, meaning that each taxon in the merged feature space occurred in at least 30% of the datasets (rounded up to the nearest integer). Relevant metadata columns were also renamed to achieve a common ontology. The merged count tables per compartment and across studies are available and documented in our repository. The number of genus-level features in each study is given in Supplementary Table S4.

#### 2.3.2 Batch effect correction and differential abundance analysis

To generate a drought signature for each compartment (endosphere, rhizosphere, bulk soil), we used the merged datasets from the nine drought studies, with features occurring in 30% of datasets (as described just before). Batch correction was performed on these merged datasets, with watering treatment as a covariate, followed by differential abundance analysis (with a log-transform), using MMUPHin v0.9 (Ma et al., 2022). To validate the signature in each individual study, we used MaAsLin2 (Mallick et al., 2021) on each study (with no prior batch correction) with log-transformed features and no filtering. The differential abundance analysis for inoculated samples was also done using MaAsLin2 on each of the four inoculation studies (with no prior batch correction), with log-transformed features, no filtering and the watering treatment (drought vs. control) as a random effect. Other parameters were set to default values.

### 2.4 Plant hosts

The merged dataset across the 13 studies consisted of 52 plant hosts. For some of the analysis, we grouped them into five clades (malvids, fabids, asterids, BOP clade, and PACMAD clade), as listed in Supplementary Table S5.

## 3 Results

### 3.1 A data integration pipeline for root and soil drought microbiome studies

To characterize the bacterial drought microbiome across different plant species and relate key drought taxa to inoculation success, we performed a meta-analysis including 13 factorial studies (Figure 1). Of these, nine investigated only one factor of interest, namely watering treatment, while the other four also included inoculation as an additional factor (Figure 1A and Supplementary Table S1). We processed amplicon reads from each study using QIIME2 (Bolyen et al., 2019), following four steps: downloading, adapter trimming, amplicon sequence variant detection, and taxonomic classification. For some studies, we also applied FIGARO (Sasada et al., 2020) to search for optimal trimming parameters to pass on to DADA2 (Figure 1A and Supplementary Table S3). In downstream processing for the drought studies, we separated count tables from our drought datasets into samples originating from bulk soil, the rhizosphere, and the endosphere. Per compartment, for the signature analysis, we only retained taxa occurring in at least 30% of studies. We then processed sample sets separately using MMUPHin (Ma et al., 2022), removing batch effects and generating a “drought signature,” consisting of taxa that were differentially abundant under drought across different plant species and studies. Lastly, we linked these key taxa to those in the inoculation studies (Figure 1A).

In total, the dataset integrates 13 studies comprising more than 3,000 samples from 52 plant hosts and bulk soil, spanning over 1,800 microbial genera, with comparable numbers of control and drought samples (Figure 1B). We observed substantial taxonomic overlap among compartments. Nearly 1,100 genera (59% of all detected) were shared across bulk soil, rhizosphere, and endosphere samples (Figure 1C), providing a common taxonomic basis for cross-compartment comparisons and suggesting the presence of a core microbiome spanning host-associated and soil environments. Rarefaction curves reveal that rhizosphere and bulk soil harbor more diverse bacterial communities than the endosphere (Figure 1D). None of the curves reaches full saturation, indicating that with 1,089 endosphere samples, 1,432 rhizosphere samples, and 761 bulk soil samples, genus-level diversity of the root and the soil was not fully captured.

### 3.2 Large-scale dataset reveals patterns in microbial composition across studies and plant hosts

**Figure 2:**
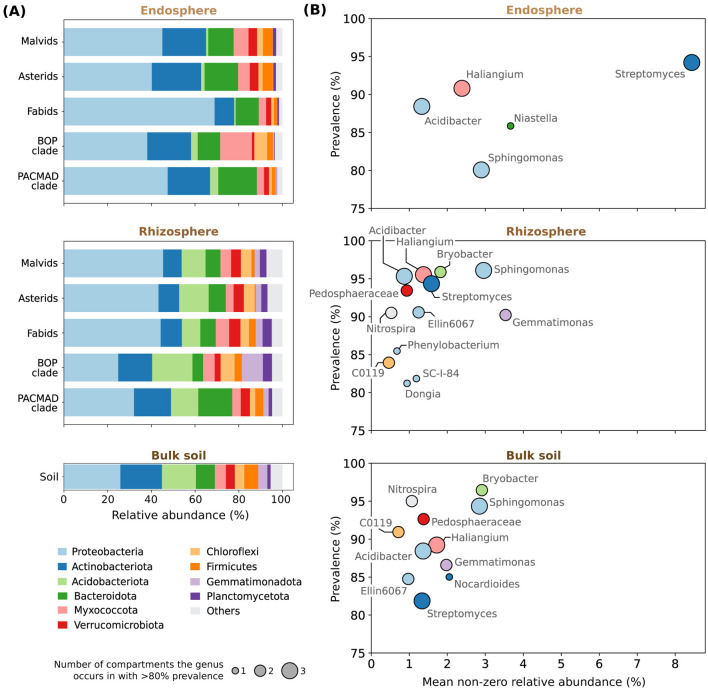
Characterization of taxonomic composition across 13 studies (Bandopadhyay et al., 2024; Santos-Medelĺın et al., 2017, 2021; Azarbad et al., 2020, 2022; Simmons et al., 2020; Xu et al., 2018; Naylor et al., 2017; Fitzpatrick et al., 2018; Moore et al., 2023; Zhang et al., 2022; Swift et al., 2025; Munoz-Ucros et al., 2022). **(A)** Phylum-level taxonomic profiles of five plant clades and bulk soil. We show the ten most abundant phyla, based on mean relative abundance across all samples. For each phylum and host (plant clades or soil), we report the mean relative abundance across samples. **(B)** Per compartment, mean non-zero relative abundances (*x*-axis) and prevalence (*y*-axis) of core genera. We only include genera that are more than 80% prevalent per compartment. Genera are also shaded based on the phylum they belong to, and different dot sizes indicate the number of compartments in which the genus occurs with over 80% abundance.

Our analysis of high-level taxonomic composition revealed that phylum abundances are characterized by compartment-specific shifts (Figure 2A). We included detailed compartment-wise differential abundance analyses on batch-corrected phylum counts in Supplementary Table S6. Significant shifts occur per compartment (*p*-value < 0.05, adjusted with Benjamini–Hochberg correction), despite considerable compositional overlap (Figure 1C). While *Bacteroidota* appear in higher abundances in the endosphere, *Planctomycetota, Gemmatimonadota*, and *Acidobacteriota* are more abundant further from the plant, in the rhizosphere and in bulk soil.

For a more fine-grained view of the endosphere and rhizosphere microbiomes, which are host-specific, we grouped the plant hosts in our dataset into five clades. This grouping accounts for hosts represented by low sample sizes and facilitates visualization of higher-level host-associated patterns. We visualized the relative abundances (prior to batch correction) of the ten most abundant bacterial phyla per clade in Figure 2A. We note several clade-specific patterns. For instance, in the endosphere, *Proteobacteria* have higher relative abundance in fabids than in other clades, supported by differential abundance analyses using batch-corrected counts (Supplementary Table S7; *p* < 0.05 for all pairwise comparisons). *Myxococcota* are similarly enriched in the endosphere of the BOP clade relative to fabids and the PACMAD clade (Supplementary Table S7). In the rhizosphere, *Firmicutes* are depleted in asterids compared to fabids, BOP, and PACMAD clades (Supplementary Table S8).

To assess taxonomic consistency across hosts and studies, we identified genera occurring at high prevalence across samples (Figure 2B). We defined these as “core” microbes, as they are present in most samples across hosts and experimental conditions. For a prevalence threshold of 80%, the endosphere contains the fewest core genera, suggesting stronger host filtering and greater variability in microbial composition relative to rhizosphere and bulk soil communities. *Streptomyces* dominate the endosphere core, with the highest mean non-zero relative abundance among all core microbes and compartments. Many core taxa are common across compartments, with *Streptomyces, Haliangium, Acidibacter* and *Sphingomonas* prevalent in all three, in more than 80% of samples. Bulk soil shares nearly all of its core microbes with the rhizosphere, with the exception of the *Actinobacteria* genus *Nocardioides*; the endosphere includes *Niastella* as a unique core microbe, while the rhizosphere core community is the largest, with three *Proteobacteria* genera emerging as unique rhizospheric core microbes.

We additionally looked at the number of prevalent microbes across multiple thresholds within each plant host clade, separately for the endosphere and rhizosphere (Supplementary Table S9). Consistent with the global, compartment-level patterns, all clades contained more core microbes in the rhizosphere than in the endosphere across all thresholds. This reinforces the existence of stronger host-specific filtering in endospheric communities, likely occurring at the plant species level rather than the broader clade scale in our grouping. Core microbe counts were also higher in host clades with smaller sample sizes.

### 3.3 Across plant hosts and experimental conditions, microbial communities reveal a shared drought footprint

**Figure 3:**
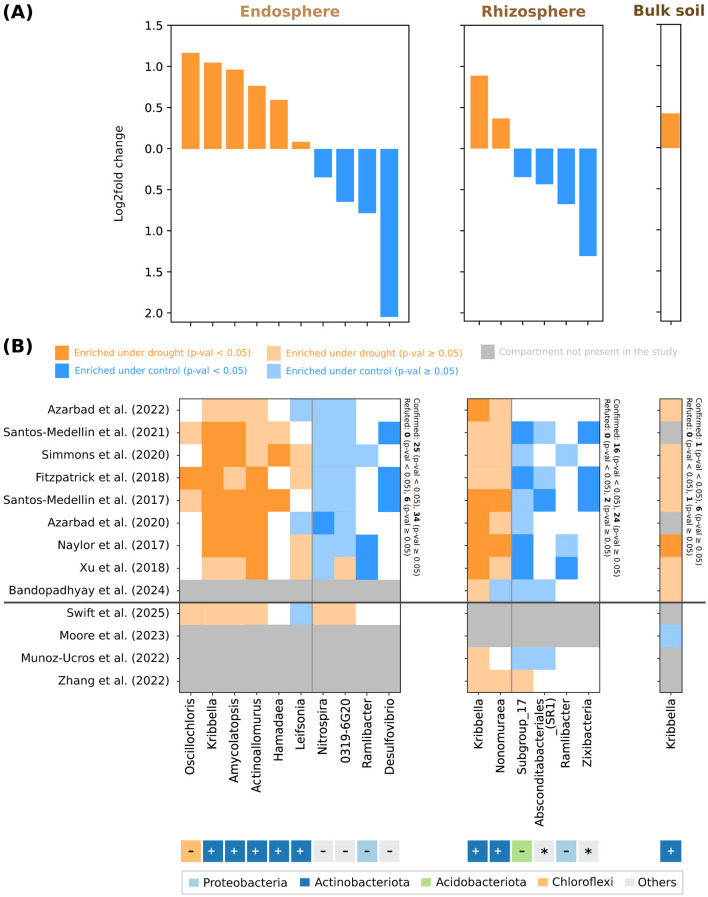
Drought signature analysis: microbes enriched and depleted under drought. Analysis was performed after batch correction, and based on adjusted *p*-values. Only features occurring in 30% of studies (per compartment) were included in the analysis. **(A)** Per compartment, the log2fold change of microbial genera differentially abundant under drought (*p*-value < 0.05). **(B)** Per-study (Bandopadhyay et al., 2024; Santos-Medelĺın et al., 2017, 2021; Azarbad et al., 2020, 2022; Simmons et al., 2020; Xu et al., 2018; Naylor et al., 2017; Fitzpatrick et al., 2018; Moore et al., 2023; Zhang et al., 2022; Swift et al., 2025; Munoz-Ucros et al., 2022) validation of the drought signature. White squares indicate that the taxon is not present in the study. The last four studies were not used for the creation of the signature in sub-figure **(A)**, and they are included for validation purposes. We mark each taxon based on phylum (coloring) and phylum Gram-stain: (+) Gram-positive, (–) Gram-negative and (*) unknown.

We next generated a drought signature consisting of bacteria differentially abundant under drought across studies (Supplementary Tables S10–S12 include all results for the differential abundance analysis). Despite being the least diverse compartment in total number of genera (Figure 1D), the endosphere contains the largest number of signature taxa, with ten showing significant shifts (*p*-value < 0.05), compared to six in the rhizosphere and just one in bulk soil (Figure 3A). The strength of drought effects also declines with distance from the plant: communities closest to the host exhibit both more affected genera at high significance values (*p*-value < 0.05), as well as larger log-fold changes. Across all compartments, *Kribella* appears enriched under drought, while *Ramilbacter* is depleted in both the endosphere and rhizosphere (Figure 3A).

An additional comparative analysis of Shannon diversity between drought and control samples (Supplementary Table S13) showed a significant difference in richness for endosphere samples: *p*≈ 0.004 and, on average, ~5% less diverse in drought. This was not the case for rhizosphere or bulk soil. When analyzed within individual plant host clades, these differences were not always significant, and drought samples were not consistently less diverse than control samples, nor vice versa. Given these variable patterns in diversity across compartments and hosts, we next checked the consistency of specific enrichment and depletion patterns at the genus level.

To test the robustness of these patterns, we examined each study individually using MaAsLin2 (Mallick et al., 2021) on the raw data counts, with no batch correction (Figure 3B). All results for the validation underlying Figure 3B are given in Supplementary Table S14. We additionally validated the differentially abundant taxa using four inoculation studies (Moore et al., 2023; Zhang et al., 2022; Swift et al., 2025; Munoz-Ucros et al., 2022), which were not used to generate the signature. We note that these studies also include inoculation as a confounder for the differential abundance analysis between watering treatments.

Most enrichment and depletion patterns are confirmed by log-fold changes in individual datasets, with no significant exceptions (*p*-value < 0.05). In the endosphere and rhizosphere, approximately 40% of the observed patterns are confirmed and significant, and, across all compartments, more than 85% are confirmed. Study-specific exceptions occur for an uncultured member of the class *Oligoflexia* (genus 0319-6G20), *Nitrospira* and *Leifsonia* in the endosphere; for *Nonomuraea* and *Acidobacteria* subgroup 17 in the rhizosphere; and for *Kribella* in bulk soil. In bulk soil, enrichment of *Kribella* is not confirmed by the external validation performed using the study of Moore et al. (2023), but, in the endosphere and rhizosphere, most enrichment and depletion patterns are also present in external inoculation studies.

The divide between enriched and depleted taxa also mirrors that between Gram-negative and Gram-positive taxa, with one exception: *Chloroflexi* genus *Oscillochloris* (Figure 3B). Notably, this is also a bacterium with a monoderm cell-wall structure, akin to that of Gram-positive bacteria. Aside from this one genus, all enrichment patterns belong to members of the Gram-positive *Actinobacteria* phylum.

### 3.4 Relation between drought signatures and inoculation response

Lastly, we examined whether drought signature taxa overlap with microbial shifts under inoculation. Based on inoculation treatment (as opposed to our previous analysis, which focused on watering treatment), we identified differentially abundant taxa in four inoculation studies (Moore et al., 2023; Munoz-Ucros et al., 2022; Swift et al., 2025; Zhang et al., 2022) and compared them with our previously generated drought signatures (Figure 3A). In rhizosphere samples from Zhang et al. (2022) and bulk soil samples from Moore et al. (2023), one drought signature genus—*Nitrospira*—was significantly depleted. We previously found that *Nitrospira* was also depleted under drought, but only in the endosphere (Figure 3).

**Figure 4:**
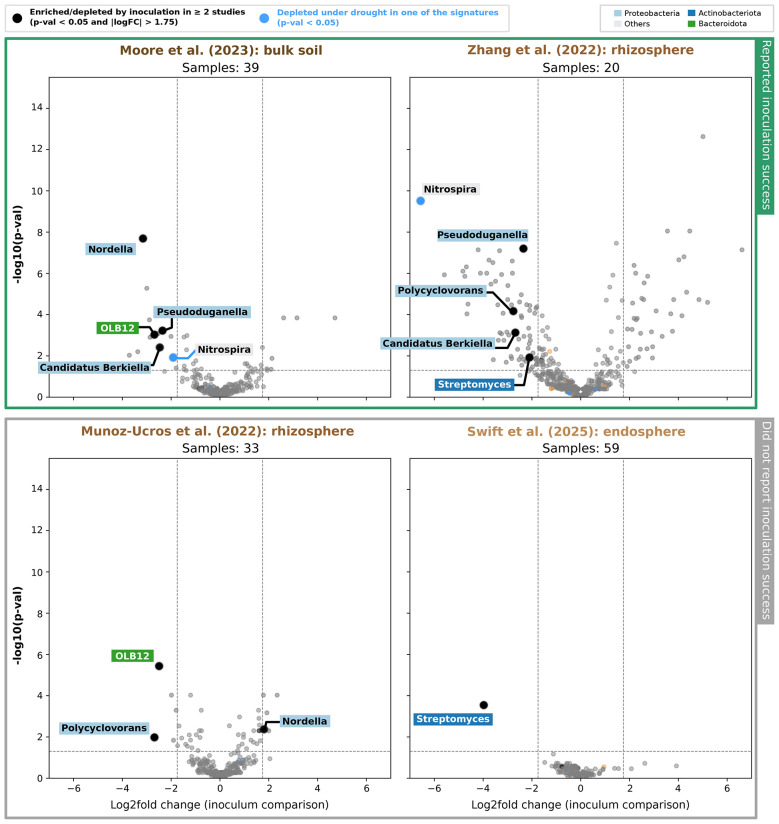
Differential abundance analysis of inoculation effects in four studies (Moore et al., 2023; Zhang et al., 2022; Swift et al., 2025; Munoz-Ucros et al., 2022). We compared dry and reference inocula, selected per study as explained in Supplementary Table S1. Taxa previously identified as being part of one of the three drought signatures (Figure 3) are marked using appropriate colors. We also mark taxa differentially abundant under inoculation in more than two of the four studies. Taxa are additionally shaded based on phylum. Horizontal lines are drawn for *p*-value = 0.05 and ∣logFC∣ = 1.75.

As shown in the upper half of Figure 4, we found inoculation to produce more pronounced effects in the two studies (Moore et al., 2023; Zhang et al., 2022) where plant performance under drought was reported as improved; we call these experiments “successful.” In contrast, the two studies reporting unsuccessful inoculations (i.e., no improvements in plant growth under drought) (Swift et al., 2025; Munoz-Ucros et al., 2022) show limited microbial responses, with fewer taxa identified as significant (*p*-value < 0.05 and ∣logFC∣ > 1.75). We also did not observe the same pattern relating magnitude of community shifts to root compartments and bulk soil as we did in our drought signature analysis (Figure 3). In fact, genera in the endosphere dataset (Swift et al., 2025) appear as the least affected by inoculation.

In addition to drought signature taxa, we also highlighted taxa that were differentially abundant under inoculation in more than two of the four studies (Figure 3). Most of these taxa are *Proteobacteria*, and they are generally depleted under inoculation. Consistent with this pattern, all taxa meeting these criteria were Gram-negative and depleted by the dry inoculum, with two exceptions. One is *Nordella*, which, though Gram-negative, was enriched in rhizosphere samples from the study of Munoz-Ucros et al. (2022). The other is *Streptomyces*, a Gram-positive genus depleted under inoculation in two of the studies. Aside from a depletion of *Nitrospira, Pseudoduganella* and a candidate genus (*Candidatus Berkiella*) in the two studies that reported successful inoculation experiments, we did not identify consistent patterns linking responses of certain microbial genera with inoculation success.

## 4 Discussion

We set out to determine whether genus-level microbial patterns of differential abundance under contrasting watering treatments generalize across studies and plant hosts. By compiling datasets from multiple studies, plant hosts, and compartments (endosphere, rhizosphere, bulk soil), we detected a drought signature consisting of genera with reproducible enrichment and depletion patterns under water limitation. This is, to our knowledge, the first meta-analysis of its kind for the drought microbiome, integrating over 3,000 samples from nine studies, as well as external validation on four separate studies. To accompany this analysis, we compiled and published a unified drought dataset covering root and soil microbiomes from all 13 studies, processed using a standardized analysis pipeline.

Our drought signature is smaller in size compared with human microbiome disease signatures identified in large-scale meta-analyses. For example, Sun et al. (2024) found 277 disease-related species across more than 6,300 gut samples, while (Li and O'Toole 2024) reported 273 species across more than 24,000 samples and multiple body sites. Both studies relied on species-level resolution, while we worked with genus-level profiles from root and soil data. These environments are harder to characterize. Plant roots and soil harbor more complex and diverse communities than the human microbiome (Anthony et al., 2023), and, so far, efforts to characterize and document them in reference databases have not sufficed, especially at the species- and strain-level (Anthony et al., 2024; Edwin et al., 2024). Sample sizes across plant hosts likely affect these patterns in microbial behavior, reflecting the greater diversity and host specificity of soil and plant-associated microbiomes compared to humans, and the need for larger and more balanced sampling across hosts. Accordingly, the smaller endosphere core microbiome points to strong species-level host filtering, while uneven host representation increased core counts in underrepresented plant clades (Section 3.2). Previous studies also documented comparable trends of host-specific microbial communities (Jackrel et al., 2021) and greater community specificity closer to the plant root (Xiong et al., 2021). Notably, we used the term “core microbiome” in a prevalence-based sense, referring to taxa consistently detected across a high percentage of samples, rather than implying functional importance.

Shannon diversity in our dataset showed compartment-specific responses to drought, although the direction of diversity changes between drought and control groups was not consistent. Endosphere communities were ~5% less diverse under drought, whereas rhizosphere and bulk soil showed no significant differences (*p*-value ≥ 0.05). We can place these results in the context of mixed observations from the studies that we integrated: some studies report no significant shifts in the same compartments (Simmons et al., 2020), while others observed stronger reductions in endosphere and rhizosphere diversity (15%–27%) under drought (Xu et al., 2018; Fitzpatrick et al., 2018).

One source of variability across studies arises from heterogeneity in how drought stress is defined. While we distinguished between drought and control conditions, differences in drought intensity and duration were not modeled, as these parameters vary widely across experiments. Drought treatments range from complete withholding of irrigation (Xu et al., 2018; Simmons et al., 2020) to protocols based on soil water holding capacity (Azarbad et al., 2020), and drought duration is reported either as days under stress (Santos-Medelĺın et al., 2021) or using coarser weekly timeframes (Naylor et al., 2017).

However, the enrichment and depletion patterns that we found align well with literature describing plant-associated responses to drought. We note that the drought-responsive microbiome is conceptually different from the core microbiome discussed previously: genera consistently present across samples are not necessarily drought-responsive, and drought-responsive genera are not necessarily part of the core microbiome. In the endosphere and rhizosphere, the drought signature is dominated by enrichment of Gram-positive taxa, particularly *Actinobacteria*, and depletion of Gram-negative taxa, a pattern observed in several studies (Xu et al., 2018; Simmons et al., 2020; Bouskill et al., 2013; Naylor and Coleman-Derr, 2018). Many of the enriched genera in our dataset belong to *Actinobacteria*. *Kribella*, in particular, is enriched across all three root and soil compartments. *Oscillochloris*, enriched in the endosphere, is an exception: although Gram-negative, it belongs to a monoderm lineage with atypical cell wall properties. This supports the argument that enrichment patterns under drought may be driven less by the Gram-positive/Gram-negative divide and more by cell wall characteristics (Xu et al., 2018).

As a case study for our drought signature, we compared enrichment and depletion shifts under drought with those present in four inoculation studies. Our meta-analysis identified only *Nitrospira*, a nitrite-oxidizing genus (Fujitani et al., 2014), as a common denominator between inoculation and drought shifts. This overlap occurred in the two successful inoculation experiments (Zhang et al., 2022; Moore et al., 2023) (Figure 4). The depletion pattern of this genus, combined with the lack of studies using it for inoculation in drought-stressed plants, indicates that it likely responds indirectly to community-level changes driven by the inoculated strains.

Although inoculation success may be context-dependent and may not rely on reproducible taxon-level changes, the stronger community shifts observed in successful studies suggest that microbiome restructuring contributes to inoculation success. Studies reporting improved plant performance under inoculation coincided with greater disruption of drought signature taxa (measured with regard to log-fold change magnitudes and significance in the volcano plots in Figure 4). To an extent, this pattern parallels results from other inoculation studies: for instance, bacterial consortia inoculation in maize altered both beta-diversity and functional composition of rhizosphere communities (Francioli et al., 2025), and inoculation in wheat under nutrient-limited conditions increased rhizosphere selection effects and the number of enriched taxa (Garrido-Sanz et al., 2023). But we have to draw these parallels cautiously, since these studies do not explicitly link log-fold changes and *p*-values in the inoculation differential abundance analysis to successful inoculation outcomes.

Interestingly, we also observed that inoculation responses contrasted with drought responses, to some extent; whereas endosphere taxa showed the strongest responses to drought, they were least affected by inoculation in the only study that measured endosphere communities (Swift et al., 2025). This contrast suggests that inoculation and drought act through distinct ecological filters, with drought more strongly affecting host-associated communities, while inoculation effects appear largely external or harder to detect.

Increased data availability and resolution may help further bridge the gap between drought and inoculation responses, advancing predictive understanding of plant–microbiome interactions under stress. Inoculation studies included relatively few samples (20–59), and, in some cases, plant measurement data was also incomplete or unavailable. In spite of data scarcity and batch effects, we obtained a robust, conservative signature by performing batch effect correction and validating signature taxa in each independent study. To expand our findings, the design of future inoculation experiments should prioritize testing under field conditions, and follow more standardized and open metadata protocols. Importantly, beneficial effects reported by inoculation studies are generally strain-specific (O'Callaghan et al., 2022), meaning that genus-level analyses will often miss them. Higher taxonomic resolution through shotgun, strain-level metagenomic analyses will strengthen the certainty of the connection between community shifts and functional traits linked to drought resilience.

## Data Availability

This study used public amplicon sequencing data, for which all accessions are documented in Supplementary Table S1. Processed count tables per study can be downloaded from our GitHub repository: https://github.com/AbeelLab/drought-microbiome-analysis.

## Footnotes

1 Available for download at this link (last accessed on 11.07.2025).
